# Parsimonious Optimization of Multitask Neural Network Hyperparameters

**DOI:** 10.3390/molecules26237254

**Published:** 2021-11-30

**Authors:** Cecile Valsecchi, Viviana Consonni, Roberto Todeschini, Marco Emilio Orlandi, Fabio Gosetti, Davide Ballabio

**Affiliations:** Department of Earth and Environmental Sciences, University of Milano-Bicocca, Piazza della Scienza 1, 20126 Milano, Italy; viviana.consonni@unimib.it (V.C.); roberto.todeschini@unimib.it (R.T.); marco.orlandi@unimib.it (M.E.O.); fabio.gosetti@unimib.it (F.G.); davide.ballabio@unimib.it (D.B.)

**Keywords:** neural networks, optimization, genetic algorithms, grid search, random search, tree-structured Parzen estimator

## Abstract

Neural networks are rapidly gaining popularity in chemical modeling and Quantitative Structure–Activity Relationship (QSAR) thanks to their ability to handle multitask problems. However, outcomes of neural networks depend on the tuning of several hyperparameters, whose small variations can often strongly affect their performance. Hence, optimization is a fundamental step in training neural networks although, in many cases, it can be very expensive from a computational point of view. In this study, we compared four of the most widely used approaches for tuning hyperparameters, namely, grid search, random search, tree-structured Parzen estimator, and genetic algorithms on three multitask QSAR datasets. We mainly focused on parsimonious optimization and thus not only on the performance of neural networks, but also the computational time that was taken into account. Furthermore, since the optimization approaches do not directly provide information about the influence of hyperparameters, we applied experimental design strategies to determine their effects on the neural network performance. We found that genetic algorithms, tree-structured Parzen estimator, and random search require on average 0.08% of the hours required by grid search; in addition, tree-structured Parzen estimator and genetic algorithms provide better results than random search.

## 1. Introduction

In recent years, due to advances in the field of artificial intelligence, the application of deep learning methodologies has rapidly spread into chemical modeling, providing new approaches to cheminformatics data. These approaches have been demonstrated to be able to successfully deal with non-linear relationships in the data and to handle multitask issues, when multiple experimental responses are considered at the same time, thus exploiting the correlation between the modeled endpoints [1,2,3].

However, compared to simpler machine learning approaches, deep learning requires more training time and depends on several hyperparameters, which affect the results and thus need to be tuned. In deep learning modeling, a major task is related to the choice of network architecture, that is, the selection of the optimal hyperparameters. Small variations in the neural network hyperparameters can frequently have a straight influence on the modeling performance [4]. Consequently, for deep learning approaches that require large training times, optimization of hyperparameters can be computationally expensive [5,6].

Common approaches for architecture optimization are grid search (GS) and random search (RS) [7]. The latter is based on random sampling among all possible architectures and thus does not guarantee achieving the optimum, while GS basically makes an exhaustive evaluation of all possible architectures by combining the selected levels of hyperparameters. Therefore, GS ensures reaching the optimal set of hyperparameters, but it is not often computationally feasible as it requires a large amount of time and resources, especially for big data and/or deep learning [8,9]. Moreover, both approaches do not provide any direct insight into the influence of hyperparameters on the architecture performances and are completely uninformed by past evaluations.

When dealing with architecture optimization in low-data regime, that is, keeping the computational requirements as low as possible, one option to obtain reliable solutions are the approaches based on artificial intelligence such as genetic algorithms (GAs) [10], which mimic the natural selection of a population of chromosomes, and tree-structured Parzen estimator (TPE) [5], which is a Bayesian hyperparameter optimization. These approaches have been used in many contexts for optimization problems [11,12,13,14,15].

In this study, we compared these four different strategies based on their ability to find the optimal set of hyperparameters for multitask neural networks. Other approaches such as gradient-based optimization [16] and multi-fidelity optimization algorithms (e.g., Hyperband) [17] were not included in this study. For a recent review on hyperparameter optimization, see [18].

The comparison was performed on three Quantitative Structure–Activity Relationship (QSAR) datasets, where the predicted endpoints were related to (i) nuclear receptors modulators (30 tasks) [19]; (ii) toxicity measurements (12 tasks); and (iii) drug annotations (two tasks) [20]. In particular, GS and RS were evaluated against GA and TPE by taking into account the performance of neural networks as well as training time and computational resources. Indeed, the results were evaluated in the context of low-data regimes, that is, looking for approaches that can provide an optimal set of solutions with the minimum number of trials, thus decreasing training efforts. Moreover, design of experiments (DoE) [21] was used to establish quantitative and qualitative relationships between the tested levels of hyperparameters and the performance of neural networks and to ensure that these were well represented in the solutions found by the optimization approaches and, in particular, the GA.

## 2. Results

### 2.1. Grid Search and Convergence of GA and TPE

The ability of GA and TPE to converge toward architectures that represent optimal or near-optimal solutions was evaluated by comparing the results of GA (10 chromosomes evolved over 42 generations) and TPE (100 trials) with the exhaustive results provided by a grid search (GS) for the three datasets.

To better compare the GA and GS solutions, we considered not only the 10 chromosomes included in the final GA population, but also the other 90 architectures tested during the generations, for a total of 100 solutions.

**Figure 1:**
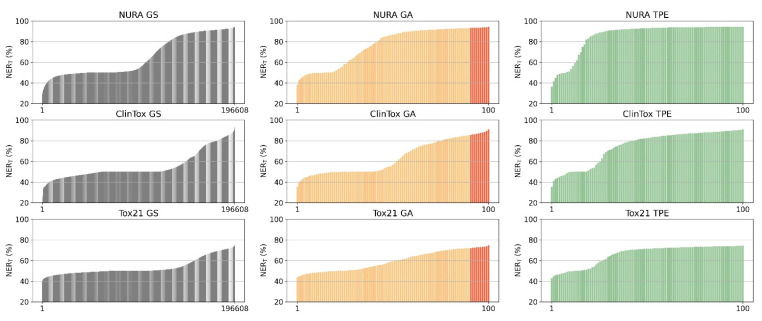
Ordered distribution of the overall non-error rate (3-fold cross-validation) of the architectures found by the optimization methods (columns, grid search, GS, genetic algorithm, GA, and tree-structured Parzen estimator, TPE) for NURA, ClinTox, and Tox21 datasets (rows). For GA, red and orange bars represent the performance of the final population (10 chromosomes) and all the other tested architectures, respectively.

As shown in Figure 1, the overall non-error rate (NER_T_) associated with the architectures generated by all the possible combinations of hyperparameters at the considered levels (i.e., GS results, 196,608 combinations) has peculiar distributions for each dataset. In particular, the NURA dataset seems to be easier to model with a half of the architectures (46%) providing a NER_T_ greater than 60%, while for the Tox21 and ClinTox dataset, this fraction was reduced 21% and 25%, respectively.

GA and TPE take on average 0.08% of the computational time required by GS, which decreases from more than a month to a few hours of calculations (Table 1). Indeed, running GS is time-consuming: without parallelization, it takes 1205 h for the smallest dataset (ClinTox) and 4905 h for the biggest one (NURA) with processors: 2 × 24-cores Intel Xeon 8160 CPU at 2.10 GHz, Cores: 48 cores/node RAM: 192 GB/node of DDR4.

GA (second column in Figure 1) was shown to be able to converge after a few generations starting from an initial population of ten chromosomes. The difference between the NER_T_ for GA and the best GS solution was between 1% (for NURA) to 3% (for ClinTox) (Table 1). TPE (third column in Figure 1) seems to explore the hyperparameter space more effectively than GA, yielding satisfactory results for most trials. However, the best TPE combinations provide NER_T_ comparable to GA, with an average difference always less than 0.5%.

Comparing the performance obtained by GS, GA, and TPE optimization (Figure 1 and Table 1), it can be noted that GA and TPE were able to converge to near-optimal solutions with only 100 total trials, thus significantly reducing computational time. TPE requires slightly longer computation time than GA and RS; this is especially apparent for the NURA dataset.

### 2.2. Random Search Vs. GA and TPE

Since GA, RS, and TPE are affected by chance, to understand whether the best solutions provided by the three approaches are significantly different, we tested the variability associated with these two methods calculating ten independent runs for RS, GA, and TPE, exploring 100 architectures per run. GA and RS are comparable in terms of required computation time (as shown in Table 1).

**Figure 2:**
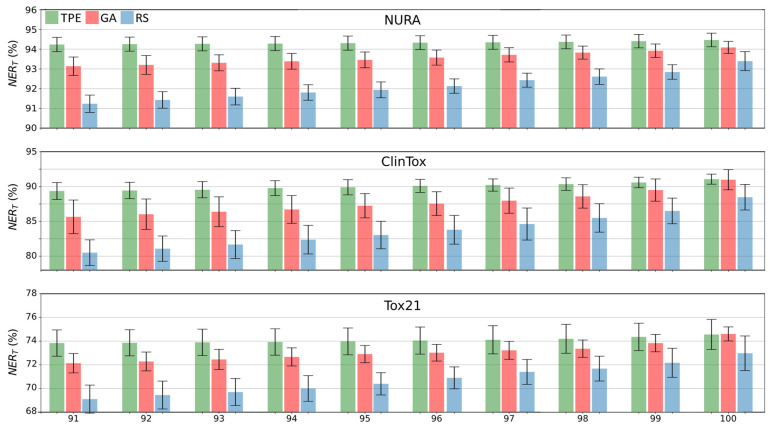
Overall non-error rate (NER_T_) of the best 10 solutions for each dataset and optimization method (tree-structured Parzen estimator, TPE, genetic algorithms, GA, and random search, RS, in green, red, and blue, respectively). Error bars are calculated considering the variability over 10 replicas.

Looking in detail at the top ten results for GA, TPE, and RS (Figure 2), it can be noted that the differences were less evident in the first rank (i.e., in the architecture associated with the highest classification performance (the three bars on the right in Figure 2)), while differences increased as the rank position decreased. It is worth noting that while the GA bars in Figure 2 represent the final population, the TPE and RS bars represent the top ten solutions after sorting the 100 trials based on NER_T_.

Furthermore, considering the standard deviation among the replicas (error bars in Figure 2), it can be concluded that the TPE and GA results are more consistent with respect to RS (i.e., their error bars are slightly smaller).

The *t*-test to compare the mean values (with a level of confidence of 95%) for all the datasets considered (see Appendix, Table A1, Table A2 and Table A3 for GA vs. RS, TPE vs. GA, and TPE vs, RS, respectively) showed that the best solutions found by GA and TPE were always significantly different from those provided by RS in terms of NER_T_. Among them, GA and TPE were significantly different only for the NURA dataset.

**Figure 3:**
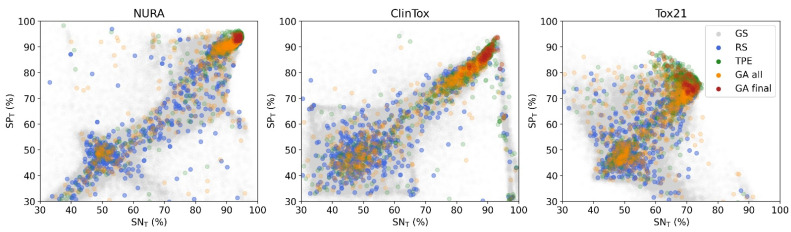
Sensitivity (SN_T_) vs. specificity (SP_T_) for the three datasets with 3-fold cross validation. Each point represents an architecture found by grid search (GS, grey points), random search (RS, light blue points), tree-structured Parzen estimator (TPE, green points), all tested architectures by genetic algorithms (GA, orange points) and architectures in the final GA populations (red points).

Hence, GA and TPE were demonstrated to provide on average better solutions than RS, that is, architectures found by GA and TPE optimization are associated with better classification performances. This can be visualized considering the overall ability to predict active and inactive compounds (i.e., overall sensitivity (SN_T_) and specificity (SP_T_), respectively), as shown in Figure 3. In all cases, two populations of solutions could be observed, one with SN_T_ and SP_T_ around 50% (worst solutions) and the other with solutions approaching those of the best GS combinations (upper right corner). GA and TPE solutions (orange and green points) converged to the upper right corner (i.e., with SN_T_ and SP_T_ values approaching 100% of correct identification). For all considered datasets, the final GA population (red points) included architectures associated with optimal classification performance.

As expected, RS solutions were randomly distributed throughout the explored space, especially in areas most populated by GS combinations (underlying grey points).

For the ClinTox dataset, an evident cluster of combinations provided increasingly unbalanced results, with SN_T_ significantly greater than SP_T_. This can be due to the extreme data imbalance in opposite directions for the two tasks, one of which had 94.1% while the other had only 7.1% of active samples in the training set.

### 2.3. Performance on External Test Set

We used the external test set to evaluate the consistency of the results provided by the considered optimization approaches. In particular, we tested the architectures obtained from the final GA populations, the best GS solution, RS, and TPE. The results were consistent with the cross-validation results (Table 2).

This time, the best solutions found by GA and TPE were significantly different from those provided by RS in terms of NER_T_ according to a t-test (at a 95% confidence level) for two of the three datasets (NURA and Tox21, see Appendix, Table A4, Table A5 and Table A6). For the ClinTox dataset, both GA and TPE results were comparable to the RS ones; indeed, all three approaches provided a NER_T_ 9% lower on average than the best GS result. For the other two datasets, GA and TPE provided better results in terms of NER_T_, with only 1% and 2% differences from the best GS solution for the NURA and Tox21 datasets, respectively. GA and TPE provided comparable results in terms of NER_T_ for all datasets. In general, TPE tends to provide solutions showing a slightly higher SP_T_ and a slightly lower SN_T_ than GA.

For the NURA dataset, the GA, TPE, and GS results showed the same good ability to predict active and inactive molecules, since the average difference between SN_T_ and SP_T_ was always 2% or less.

In contrast, the best GS model on the Tox21 dataset better predicts inactive than active compounds with a difference between SP_T_ and SN_T_ around 9%.

### 2.4. GA and DoE

DoE was applied as a supporting tool to understand how hyperparameters affect the performance when varying their levels. In particular, D-optimal designs were applied in this framework. The D-optimal results were also used as a term of comparison with the results provided by GA, which were evaluated in terms of frequency of hyperparameter values in the final GA population.

**Figure 4:**
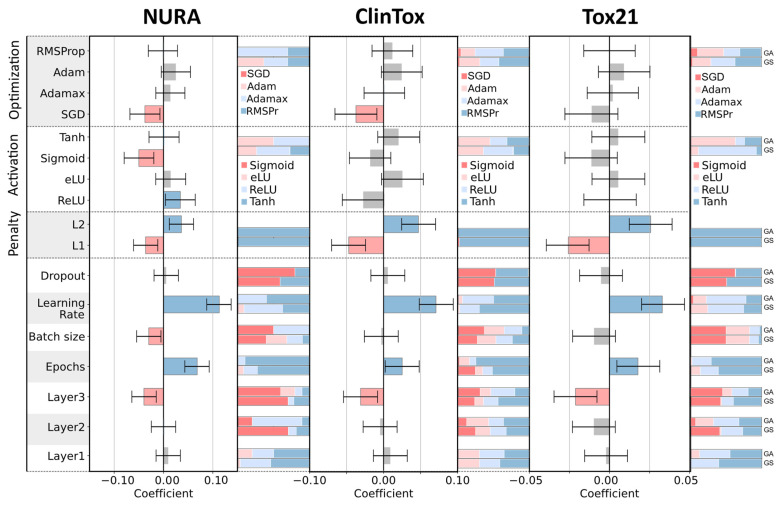
Coefficients of the D-optimal models and relative frequency bars of the hyperparameters among the genetic algorithms (GA) final population (upper bar) and the best 1000 architectures obtained with the grid search (GS) strategy (lower bar). For quantitative hyperparameters, the bars are colored according to Figure 5.

Figure 4 compares the DoE coefficient for each hyperparameter with its frequency among the architectures tested in the final GA population and among the best thousand GS solutions (top and bottom bars on the right side of the coefficient plot, respectively).

The DoE coefficients can be significantly positive or negative when the values of the corresponding hyperparameter (in case of quantitative factors) or corresponding levels (in case of qualitative factors) are associated with an increase or decrease in NER_T_. Otherwise, the importance of the hyperparameter is considered inconclusive.

**Figure 5:**
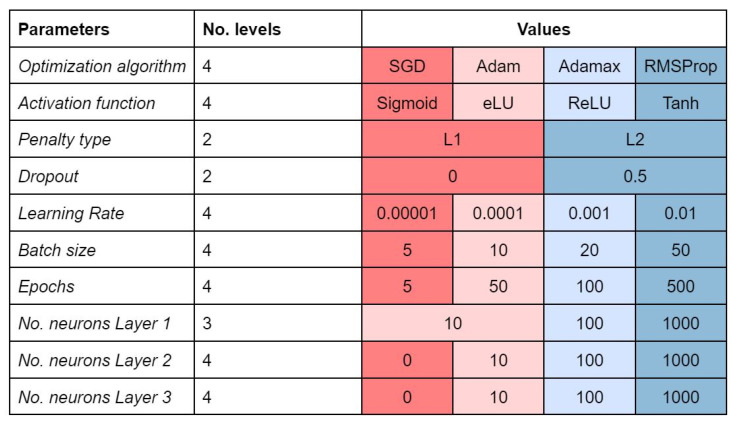
Hyperparameters to be tuned and the levels considered. Levels are colored to facilitate the comprehension of Figure 4.

In Figure 4, coefficients associated with negative, positive, and inconclusive values are colored in red, blue, and grey, respectively. The frequency bars are colored according to Figure 5 where low and high values are denoted by the colors red and blue, respectively.

The most relevant quantitative factors are learning rate (the higher the better), penalty (L2), epochs (the higher the better), and the number of neurons in the third layer (the lower the better).

The learning rate was set to high values (0.01 or 0.001) in 100%, 94%, and 78% of the architectures in the final GA population and 91%, 97%, and 75% of the architectures in the best GS solutions for NURA, ClinTox, and Tox21 dataset, respectively. This suggests that high learning rate is preferable, and that GA and GS are consistent with the DoE analysis. The same considerations can be drawn for the number of epochs.

The L1 penalty seems to be consistently associated with unsatisfying performance having a significant negative coefficient value for all three datasets. Accordingly, the architectures containing the L1 penalty (red) were almost absent in the best GA and GS solutions, as can be seen from the frequency bars that were almost entirely colored blue.

SGD, as an optimization algorithm, had a large negative coefficient value for NURA and ClinTox; sigmoid as activation function should be avoided for the NURA dataset due to its negative coefficient. Again, there was a good qualitative correspondence between the D-optimal coefficients and the GA/GS frequency bars. In particular, SGD was absent from the best GA and GS results for the NURA dataset and present only in 4% and 2% of the best GA and GS ClinTox architectures, respectively.

For the NURA dataset, the sigmoid function is present in only 0.2% of the best GS solutions and absent in the final GA population.

The number of neurons in the first two layers and the dropout value seem to be irrelevant for all datasets according to the D-optimal models; this was also confirmed by the well-balanced frequency distributions observed for both the GA and GS architectures. In this case, a better balance between low and high levels (red and blue colors) could be observed in the frequency distributions between the best GA and GS architectures.

## 3. Discussion

We performed ten independent GA, TPE, and RS runs computing a total of 100 architectures for each approach and then we compared their results with those of an exhaustive grid search (196′608 hyperparameter combinations) on three multitask QSAR datasets. With an average of 0.08% of the hours required by GS, the other approaches, RS, TPE, and GA significantly lowered the computational time required. TPE required slightly more time than GA (0.6 and 1.9 h for Tox21 and NURA, respectively).

TPE provided consistently good results, while GA was shown to converge to near-optimal results after a few generations starting from an initial population of 10 chromosomes; the difference between the average NER_T_ for the best results of GA, TPE, and GS ranged from 1% (for NURA) to 3% (for ClinTox).

Moreover, GA and TPE provided better and more consistent results than RS for all datasets in three-fold cross-validation and for two out of three external test sets. TPE provided better results for one dataset (NURA) in three-fold cross-validation, but was comparable to GA on the external test set for all datasets considered.

Comparison of DoE coefficients and parameter frequency in the GA final population showed a good match. In particular, for all the datasets, we concluded that (i) the optimal number of neurons in the third layer corresponded to the lowest level tested; (ii) the optimal number of epochs and learning rate corresponded to the highest level tested; and (iii) L2 was the best penalization strategy. In addition, we identified dataset-specific patterns; for example, SGD optimization algorithm had a significant negative coefficient value for NURA and ClinTox, whereas sigmoid as an activation function should be avoided for the NURA dataset.

Knowing the best levels of the considered hyperparameters, the GS ensures obtaining the best performance, however, if there are time and computation limitations, approaches based on artificial intelligence such as GA and TPE offer a good alternative that can achieve good performance in a limited amount of time and thus obtain a good trade-off between classification results and required resources.

## 4. Materials and Methods

### 4.1. Dataset

In this study, we used the following benchmark and freely available multitask datasets, whose main characteristics are summarized in Table 3:

1. NUclear Receptor Activity dataset (NURA) is a recently published dataset [19] containing information on nuclear receptor modulation by small molecules. Chemicals can bind to nuclear receptors by activating (agonist) or inhibiting (antagonist) the natural biological response. Hence, the NURA dataset contains annotations for binding, agonism, and antagonism activity for 15,206 molecules and 11 selected NRs. For each receptor and each activity type (agonism, binding, antagonism), a molecule can have one of the following annotations: (i) ‘active’; (ii) ‘weakly active’; (iii) ‘inactive’; (iv) ‘inconclusive’; and (v) ‘missing’. In this work, each bioactivity type for a given receptor was considered as a task. As in [22], only active and inactive annotations were considered and tasks containing such annotations for less than 200 molecules were discarded. The considered dataset is therefore composed of a total of 14,963 chemicals annotated (as active or inactive) for at least one of the selected 30 tasks.

2. Tox21 dataset contains qualitative toxicity measurements on 12 biological targets (i.e., tasks) including nuclear receptors and stress response pathways. This dataset is curated by MoleculeNet as a benchmark for molecular machine learning [20]. The 7831 compounds were pruned for disconnected structures obtaining a final dataset of 7586 molecules.

3. ClinTox dataset contains qualitative data of drugs approved by the FDA and those that have failed clinical trials for toxicity reasons (i.e., two tasks). Additionally, this dataset is curated by MoleculeNet [20]. The 1478 compounds were pruned for disconnected structures obtaining a final dataset of 1472 molecules.

Molecules of each dataset were randomly split into the training (80%) and external test set (20%), trying to preserve the proportion between the classes (actives/inactives) for each task (stratified splitting). All analyses were performed in 3-fold cross-validation (i.e., for each architecture, three neural networks were trained on three different subsets of training data and performance was evaluated on the excluded data each time).

For each molecule, we computed extended connectivity fingerprints (ECFPs) [23] as molecular descriptors and these were used as input variables for neural networks. ECFPs are binary vectors of predefined length that encode the presence/absence of atom-centered substructures through a hashing algorithm. In particular, we computed ECFPs with the following options: 1024 as the fingerprint length, two bits to encode each substructure, and a fragment radius comprised between 0 and two bonds.

### 4.2. Multitask Neural Network

The most common multitask networks in the literature are constituted by fully connected neural network layers trained on multiple tasks, where the output is shared among all learning tasks and then fed into individual classifiers. When some dependence relationships exist among the tasks, the model should learn a joint representation of these tasks and, thus benefit from an information boost [2,24]. Similar to single-task feedforward neural networks, the input vectors are mapped to the output vectors with repeated compositions of layers, which are constituted by neurons. When each neuron of a layer is connected to all the neurons of the following layer, the network is called dense or fully connected. The layers between input and output are called hidden layers. Each connection represents a weight, whereas each node represents a learning function *f* that, in the feedforward phase, processes the information of the previous layer to be fed into the subsequent layer. In the backpropagation phase, each weight is adjusted according to the loss function and the optimization algorithm. Figure 5 summarizes the hyperparameters we decided to tune.

Different types of activation functions exist in the literature; the most known are sigmoid, rectified linear unit (ReLU), hyperbolic tangent (Tanh), and exponential linear unit (eLU), which can be defined for an input *z* as follows:

(1) $$Sigmoid\left(z\right)=\frac{1}{1+e^{-z}}$$

(2) ReLU(z)=max(0,z)

(3) $$Tanh\left(z\right)=\frac{e^{z}-e^{-z}}{e^{z}+e^{-z}}$$

(4) $$eLU\left(z\right)=\{\begin{array}{r} e^{z}-1,\;z<0\\ z,\;z\geq 0 \end{array}$$

Neural network tuning implies setting a learning rate that determines the update of the weights in each iteration with respect to the gradient of the loss function. For computational and learning efficiency, in each training step (i.e., iteration), a subset of training examples called batch is used. When all the examples are seen by the model (i.e., after a number of iterations equal to the training set size divided by the batch size), a training epoch is completed.

Furthermore, several strategies called regularization techniques can improve the network’s generalizing ability and reduce overfitting. This is the case of dropout, where a percentage of randomly selected neurons are ignored during training, and the introduction of a penalty also called L1 or L2 regularization, on the basis of the chosen function.

In this work, we used the binary cross-entropy as loss function, which can handle multiple outputs and missing data. We considered both ‘shallow’ (i.e., only one hidden layer) and deep architectures up to three hidden layers, and neurons per layer varying between 0 and 1000. The output layer consists of as many nodes as tasks. The threshold of assignment for the output nodes was optimized on the basis of ROC curves for each task, that is, if the output of the neural network ensemble node is equal or lower than the threshold the compound is predicted inactive, otherwise active. We initialized the network weights randomly according to a truncated normal function with epochs varying from five to 500.

### 4.3. Classification Performance of Multitask Neural Networks

The model performance on each *t*-th task was quantified and TP_t_, TN_t_, FP_t_, and FN_t_ were computed as the number of true positive, true negative, false positive, and false negative for the *t*-th task. To compare the overall performance of models, ‘global’ sensitivity, specificity, and non-error rate measures (SN_T_, SP_T_, NER_T_) were computed as follows [25]:

(5) $${\mathrm{SN}}_{T}=\frac{\sum_{t=1}^{T}{\mathrm{TP}}_{t}}{\sum_{t=1}^{T}{\mathrm{TP}}_{t}+\sum_{t=1}^{T}{\mathrm{FN}}_{t}}\cdot 100$$

(6) $${\mathrm{SP}}_{T}=\frac{\sum_{t=1}^{T}{\mathrm{TN}}_{t}}{\sum_{t=1}^{T}{\mathrm{TN}}_{t}+\sum_{t=1}^{T}{\mathrm{FP}}_{t}}\cdot 100$$

(7) $${\mathrm{NER}}_{T}=\frac{{\mathrm{SN}}_{T}+{\;\mathrm{SP}}_{T}}{2}\cdot 100$$

where t runs over each task, and T is the total number of tasks. SN_T_ and SP_T_ represent the percentage of active and inactive molecules correctly predicted over all tasks, respectively.

### 4.4. Optimization Strategies

We compared three well-known strategies to tune the network hyperparameters: grid search, random search and genetic Algorithms [8].

Grid search (GS) is the most computationally expensive and time-consuming approach since it explores all the possible combinations of hyperparameters at the selected levels in the search space. In our case study, 196,608 architectures were tested. GS suffers from high dimensional spaces, but can often be easily parallelized.

Random search (RS) strategy avoids the complete selection of all combinations by a random selection of combinations and is more efficient than GS [7]. We chose to randomly select a subset of GS combinations to limit the exploration to the levels of values reported in Figure 5. The number of random combinations to test is user-defined, usually based on a trade-off between available computational time/power and satisfying performance. We chose to select a subset of one hundred combinations.

Genetic algorithms (GA) are heuristic stochastic evolutionary search algorithms based on sequential selections, combinations, and mutations simulating biological evolution. In other words, combinations of hyperparameters leading to a higher performance or fitness survive and have a higher chance to reproduce and generate children with a predefined mutation probability. Each generation consists of a population of chromosomes representing points in the search space. Each individual is represented as a binary vector. The considered algorithm consists of the following steps:

1. Randomly generate a population of 10 chromosomes;

2. Compute the fitness as overall NER (NER_T_) in 3-fold cross validation for each chromosome of the population;

3. Select two “parental” chromosomes on a roulette wheel basis (i.e., the probability to be selected is proportional to the fitness);

4. Generate two children with a recombination of the parental chromosomes and with a mutation probability for each bit equal to 10%;

5. Evaluate the fitness for the children and insert them into the population;

6. Exclude the two worst-performing chromosomes (i.e., those with the lowest NER_T_) of the population;

7. Repeat steps 3–7 for 42 times (or generations); and

8. At the 21st generation, replace the worst performing half of the population with randomly generated chromosomes (cataclysm or invasion simulation).

This process allows us to evaluate 100 combinations of hyperparameters.

Tree-structured Parzen estimator (TPE) is a sequential model-based optimization (SMBO) approach [5]. SMBO methods sequentially construct models to approximate the performance of hyperparameters based on historical measurements, and then subsequently chooses new hyperparameters to test based on this model. The TPE approach models P(*x*|*y*) and P(*y*) following the Bayesian rule, where *x* represents hyperparameters and *y* the associated quality score. P(*x*|*y*) is modeled by transforming the generative process of hyperparameters, replacing the distributions of the configuration prior with non-parametric densities. TPE consists of the following steps:

1. Define the domain of hyperparameter search space;

2. Create an objective function which takes in input the hyperparameters and outputs a score to be minimized or maximized;

3. Obtain a couple of observations (score) using randomly selected combinations of hyperparameters;

4. Sort the collected observations by score and divide them into two groups: group (*x*_1_) contains observations that gave the best scores and the second one (*x*_2_) all other observations;

5. Model two densities *l*(x_1_) and *g*(x_2_) using Parzen estimators (or kernel density estimators);

6. Raw sample hyperparameters from *l*(x_1_), evaluating them in terms of *l(x*_1_)/*g(x*_2_), and returning the set that yields the minimum value under *l(x*_1_)/*g(x*_1_) corresponding to the greatest expected improvement. These hyperparameters are then evaluated on the objective function;

7. Update the observation list from step 3; and

8. Repeat steps 4–7 with a fixed number of trials.

We set the total number of trials to 100 and the 3-fold cross-validation NER_T_ as the score to maximize. We chose predefined values for each hyperparameters in order to constrain the possible combinations to the grid search space.

RS, GA, and TPE strategies were independently repeated ten times to guarantee robustness of the results and to measure their uncertainty.

Experimental design (or design of experiments, DoE) aims at maximizing the information on an investigated system or process and at the same time minimizing the experimental effort; this is achieved by a rational experimental plan to obtain the best knowledge of the system [21]. In this framework, DoE was used to obtain quantitative relationships between architectures and the performance of multitask networks, thus, to understand how the choice of hyperparameter values can directly influence the modeling.

The D-optimal design is a particular kind of experimental design [26], which is commonly used when (i) the shape of the analyzed domain is not regular; (ii) there are qualitative factors together with quantitative ones; and (iii) the number of experimental runs has to be reduced. This was the case with the application of DoE on the hyperparameters in this case study, where both qualitative and quantitative factors were present and, being the number of considered hyperparameters large, we had to reduce the computational effort in terms of neural network architectures tested.

### 4.5. Software

ECFPs were computed using the software Dragon 7 [27] (count fragments = true, atom options: [atom type, aromaticity, connectivity total, charge, bond order]).

The multitask models were calculated in Python 3.6.4 [28] using the Keras 2.4.3 [29] package with TensorFlow 2.4.1 [30] backend. MODDE 9 Software (Umetrics, Malmö, Sweden) was used to perform the DoE analysis. TPE experiments were performed by means of Optuna 2.10.0 package in Python [14].

## Figures and Tables

**Table 1:** Results in terms of overall non-error rate (NER_T_) considering grid search (GS), genetic algorithms (GA), random search (RS), and tree-structured Parzen estimator (TPE) as optimization strategies in 3-fold cross validation. The computational time for 3-fold cross-validation is also reported in hours (h). Mean and confidence interval among 10 replicas are reported for GA, RS, and TPE results.

	Cross-Validation					
	NURA		ClinTox		Tox21	
	Best NER_T_	Time (h)	Best NER_T_	Time (h)	Best NER_T_	Time (h)
**GS**	95.1	4905.3	93.7	1205.2	76.4	2858.7
**GA**	94.1 ± 0.2	3.3	91.0 ± 1.1	1.0	74.6 ± 0.5	2.3
**TPE**	94.5 ± 0.2	5.1	91.1 ± 0.5	1.6	74.6 ± 0.8	2.8
**RS**	93.4 ± 0.4	3.2	88.5 ± 1.4	0.8	73.0 ± 1.0	2.2

**Table 2:** Results in terms of overall non-error rate (NER_T_), sensitivity (SN_T_), and specificity (SP_T_) considering grid search (GS), genetic algorithms (GA), random search (RS), and tree-structured Parzen estimator (TPE) as optimization strategies on the external test set. Confidence intervals among 10 replicas are reported for the GA and RS results.

	External Set								
	NURA			ClinTox			Tox21		
	NER_T_	SN_T_	SP_T_	NER_T_	SN_T_	SP_T_	NER_T_	SN_T_	SP_T_
**GS**	95.5	95.3	95.7	95.6	94.9	96.2	78.7	74.3	83.1
**GA**	94.4 ± 0.3	94.6 ± 0.4	94.1 ± 0.4	86.4 ± 2.9	88.8 ± 2.6	84.7 ± 4.5	77.6 ± 0.7	75.9 ± 0.8	79.3 ± 1.8
**TPE**	94.7 ± 0.3	94.5 ± 0.3	95.0 ± 0.7	86.6 ± 3.1	87.6 ± 5.3	85.5 ± 3.5	77.4 ± 0.8	75.5 ± 2.0	79.4 ± 2.4
**RS**	93.7 ± 0.6	94.1 ± 0.5	93.2 ± 0.9	86.5 ± 3.4	88.4 ± 3.1	84.7 ± 4.5	75.7 ± 1.3	74.6 ± 2.2	76.7 ± 3.6

**Table 3:** Summary information of the considered multitask datasets.

Dataset	Description	No. Tasks	No. Samples	Ref.
**NURA**	Qualitative bioactivity annotations for 11 selected nuclear receptors.	30	14,963	[19]
**ClinTox**	Qualitative data of drugs approved by the FDA and those that have failed clinical trials for toxicity reasons.	2	1472	[20]
**Tox21**	Qualitative toxicity measurements on 12 biological targets.	12	7586	[20]

## Data Availability

Data available in publicly accessible repositories: 10.5281/zenodo.3991562 (NURA dataset) and https://moleculenet.org/datasets-1 (ClinTox and Tox21), accessed on 5 October 2021.

## Sample Availability

Not available.

## Appendix A

**Table A1:** Two-tailed t-test with a hypothesized zero mean difference for the three datasets considering the best result among 10 replicas between genetic algorithms (GA) and random search (RS) on 3-fold cross validation. Variances were assumed unequal only for Tox21 (*p*-value of F test 0.007).

	NURA (GA, RS)	ClinTox (GA, RS)	Tox21 (GA, RS)
**Mean**	94.1, 93.4	91.0, 88.5	74.6, 73.0
**Variance**	0.001, 0.003	0.023, 0.037	0.004, 0.023
**Pooled Variance**	0.002	0.031	-
**Degrees of freedom**	18	18	12
**t value**	3.643	3.214	3.104
**P(t ≤ t) two-tail**	0.002	0.005	0.009
**t critical (95%)**	2.101	2.101	2.179

**Table A2:** Two-tailed t-test with a hypothesized zero mean difference for the three datasets considering the best result among 10 replicas between genetic algorithms (GA) and tree-structured Parzen estimator (TPE) on 3-fold cross validation. Variances were assumed unequal for Tox21 and ClinTox (*p*-value of F test 0.017 and 0.024, respectively).

	NURA (GA, TPE)	ClinTox (GA, TPE)	Tox21 (GA, TPE)
**Mean**	94.1, 94.5	91.0, 91.1	74.6, 74.6
**Variance**	0.001, 0.001	0.02, 0.006	0.004, 0.002
**Pooled Variance**	0.001	-	-
**Degrees of freedom**	18	13	13
**t value**	−2.466	−0.147	0.100
**P(t ≤ t) two-tail**	0.024	0.885	0.922
**t critical (95%)**	2.101	2.160	2.160

**Table A3:** Two-tailed t-test with a hypothesized zero mean difference for the three datasets considering the best result among 10 replicas between tree-structured Parzen estimator (TPE) and random search (RS) on 3-fold cross validation. Variances were assumed equal.

	NURA (TPE, RS)	ClinTox (TPE, RS)	Tox21 (TPE, RS)
**Mean**	94.5, 93.4	91.1, 88.5	74.6, 73.0
**Variance**	0.001, 0.003	0.006, 0.037	0.018, 0.024
**Pooled Variance**	0.002	0.022	0.021
**Degrees of freedom**	18	18	18
**t value**	5.441	3.935	2.454
**P(t ≤ t) two-tail**	0.004	0.001	0.024
**t critical (95%)**	2.101	2.101	2.101

**Table A4:** Two-tailed t-test with a hypothesized zero mean difference for the three datasets considering the best result among 10 replicas between genetic algorithms (GA) and random search (RS) on the external test set. Variances were assumed unequal for Tox21 and NURA (*p*-value of F test 0.04 and 0.02).

	NURA (GA, RS)	ClinTox (GA, RS)	Tox21 (GA, RS)
**Mean**	94.4, 93.7	86.4, 86.5	77.6, 75.6
**Variance**	0.002, 0.007	0.163, 0.226	0.009, 0.032
**Pooled Variance**	-	0.194	-
**Degrees of freedom**	10	18	14
**t value**	2.370	−0.084	3.024
**P(t ≤ t) two-tail**	0.034	0.934	0.009
**t critical (95%)**	2.160	2.101	2.144

**Table A5:** Two-tailed t-test with a hypothesized zero mean difference for the three datasets considering the best result among 10 replicas between genetic algorithms (GA) and tree-structured Parzen estimator (TPE) on an external test set. Variances were assumed equal.

	NURA (GA, TPE)	ClinTox (GA, TPE)	Tox21 (GA, TPE)
**Mean**	94.4, 94.7	86.4, 86.6	77.6, 77.4
**Variance**	0.002, 0.002	0.162, 0.451	0.009, 0.015
**Pooled Variance**	0.002	0.307	0.012
**Degrees of freedom**	18	18	18
**t value**	−1.852	−0.088	0.269
**P(t ≤ t) two-tail**	0.080	0.931	0.790
**t critical (95%)**	2.101	2.101	2.101

**Table A6:** Two-tailed t-test with a hypothesized zero mean difference for the three datasets considering the best result among 10 replicas between tree-structured Parzen estimator (TPE) and random search (RS) on an external test set. Variances were assumed equal.

	NURA (TPE, RS)	ClinTox (TPE, RS)	Tox21 (TPE, RS)
**Mean**	94.7, 93.7	86.6, 86.5	77.4, 75.6
**Variance**	0.002, 0.007	0.451, 0.225	0.014, 0.031
**Pooled Variance**	0.005	0.338	0.023
**Degrees of freedom**	18	18	18
**t value**	3.450	0.020	2.629
**P(t ≤ t) two-tail**	0.003	0.984	0.017
**t critical (95%)**	2.101	2.101	2.101
